# Deformation constitutive model of subgrade soil under intermittent cyclic loading

**DOI:** 10.1038/s41598-023-27502-w

**Published:** 2023-01-06

**Authors:** Bolang Zhang, Kaisheng Chen, Xing Hu, Xingwei Zhang, Guofu Luo, Rongya Chen

**Affiliations:** School of Civil Engineering, Guizhou University, Guiyang, 550025 China

**Keywords:** Environmental sciences, Solid Earth sciences

## Abstract

During the actual operation of subways, high-speed railway, and other infrastructure, subgrade soil will be subjected to periodic vibration and intermittent loading, i.e., intermittent cyclic load. To study the deformation characteristics of subgrade soil under intermittent cyclic loading, the Newtonian dashpot in the generalized Kelvin model is replaced by the fractional Abel dashpot, a fractional generalized Kelvin model is established. Then, a nonlinear viscoplastic body model considering damage is proposed, which is connected in series with the improved fractional generalized Kelvin model, and the deformation constitutive model of soil under intermittent cyclic loading is obtained. Finally, the effectiveness and applicability of the model are analyzed, and the model is compared with other constitutive models. The results show that the constitutive model established in the paper can reflect the combined effect of confining pressure, frequency, dynamic stress amplitude and other factors on the cumulative plastic strain of soil, and can well characterize the development law of three types of cumulative plastic strain curves of soil: stable, critical, and failure. The fitting parameters $\alpha$ and $\eta$ tend to decay and rise, respectively, with the increase in loading stage *N*, which reflects the rationality and accuracy of the model.

## Introduction

With the vigorous development of the economy and the continuous rise in consumption, the development of the logistics industry has gradually become one of the most important indicators used to assess a country’s modernization and comprehensive national power, which means that the carrying capacity of freight trains, freight cars, and other means of transportation has continued to rise. Subgrade, as a structure-bearing traffic load, has strength, stiffness, and other mechanical indexes that will decay with an increase in the number of traffic load cycles, resulting in excessive long-term settlement of the subgrade, which threatens the safety of infrastructure^1–3^.

Aiming to determine the action mechanism of long-term settlement of subgrade soil caused by traffic load, many scholars have carried out dynamic triaxial tests on soil samples with various mechanical properties, such as saturated soft clay^4^, silt^5^, frozen soil^6^, and compacted loess^7^, and systematically explored the influence of dynamic stress amplitude, cycle times, confining pressure, moisture content, and other factors on the dynamic characteristics of subgrade filler. For example, Cai^8^ and others took cement-improved expansive soil as the research object, pointing out that the dynamic parameter of the subgrade under the soaking state is greater than the natural state value, and subgrade soaking will promote the cumulative deformation of the subgrade. Lei and Lin^9,10^ analyzed the influence of freeze–thaw cycle times, freezing temperature, confining pressure, and other factors on the cumulative plastic strain of subgrade soil, and created an empirical model to predict the cumulative plastic strain of soil. Sun et al.^11^ studied the influence of variable confining pressure on the permanent deformation of soil, and pointed out that, when constant confining pressure and variable confining pressure tests have the same maximum stress or average stress, the variable confining pressure can accumulate more permanent strain. In order to study the influence of temperature on soil dynamic characteristics, Xiong et al.^12^ developed a temperature-controlled triaxial test device. The research results showed that, with an increase in temperature, the cumulative plastic strain, dynamic damping ratio, and dynamic pore pressure decrease. Thevakumar et al.^13^ considered the rotation of the principal stress in the subgrade, carried out a series of dynamic hollow cylinder tests, and pointed out that the rotation of the principal stress had an adverse effect on the axial cumulative strain of soil and soil degradation, with pore water pressure less affected by the rotation of the principal stress. Scholars have carried out comprehensive and in-depth research based on the fact that subgrade soil may be in different natural conditions in the actual operation process, discussing the action of variable confining pressure, temperature, principal stress rotation, and other factors on the dynamic characteristics of subgrade soil, and have made significant contributions to the theoretical and practical research on the cumulative plastic strain of subgrade soil under traffic load.

However, the abovementioned researchers did not consider the periodic loading characteristics of traffic loads on the subgrade soil when carrying out dynamic triaxial tests, and all conclusions were obtained under the condition of continuous loading. In view of this, considering that the subgrade soil is subjected to periodic vibration and intermittent repeated action under the action of traffic loads such as trains and subways, Zheng, Lei, Feng, Nie^14–17^ and others carried out dynamic triaxial tests of intermittent loading, analyzed the influential mechanism of intermittent loading, and found that the existence of an intermittent period will improve the ability of subgrade soil to hinder its strain softening; in the intermittent stage, the excess pore water pressure generated in the cyclic loading stage dissipates. The redistribution of stress between soil particles makes the soil structure and particles readjust, and the sample reconsolidates. According to the deformation characteristics of subgrade soil under intermittent cyclic load, Nie and Li^17,18^ and other scholars proposed empirical models to predict the cumulative plastic strain of fine-grained soil under intermittent cyclic loads. For the deformation characteristics of subgrade soil under continuous cyclic loading, Lin and Chen et al.^10,19^ proposed an empirical model of cumulative deformation of subgrade soil under continuous cyclic loading. However, a weakness is that the parameters in these empirical models are easily affected by the test environment, which makes them show strong instability in the process of predicting the cumulative permanent deformation of different soils. Although there are many research articles on soil deformation models, most of them focus on the deformation constitutive model of soil under continuous cyclic loading conditions, while few articles have been published on the dynamic properties of subgrade soil under intermittent cyclic loading. Therefore, in order to understand the deformation law of different subgrade soils under intermittent cyclic loading with the help of theoretical and mathematical tools, it is necessary to continue to carry out research on the constitutive model of soil deformation under intermittent cyclic loading.

In accordance with the characteristics of static deviator stress and alternating stress of subgrade soil in the actual operation, the total axial strain is divided into creep strain and strain response. By means of an element model, fractional calculus theory, and viscoplastic theory, the deformation constitutive model of subgrade soil under intermittent cyclic load is derived. Then, the applicability of the constitutive model is verified by using the dynamic triaxial test data of fine-grained soil and saturated soft clay under intermittent loading, and the parameters are analyzed. Finally, the proposed model is compared with other constitutive models, and the advantages of this one are revealed. The research results are of significance for the calculation and analysis of the long-term settlement of subgrade soil under traffic loads.

## Deformation theory of subgrade soil under intermittent cyclic load

### Intermittent cyclic load analysis

**Figure 1:**
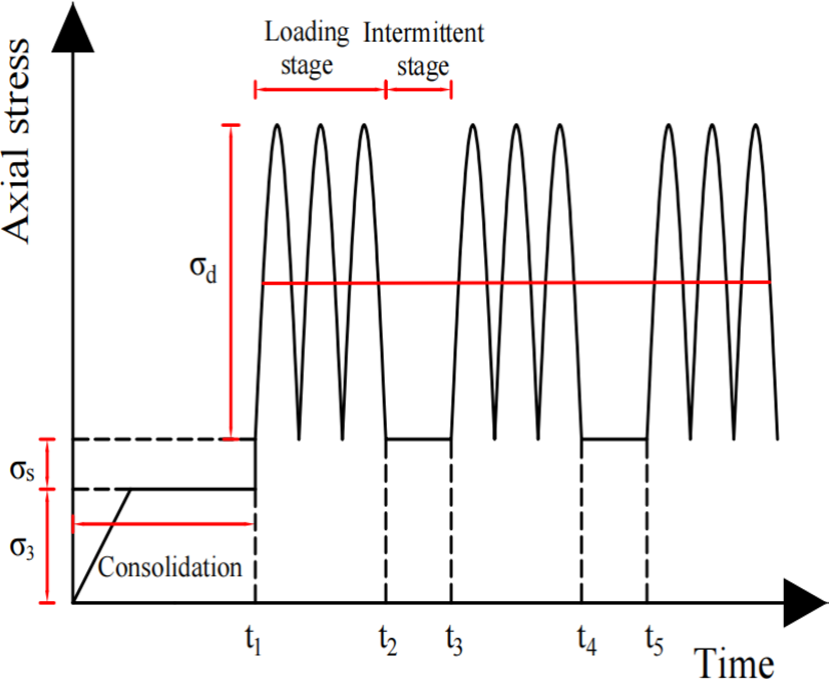
Intermittent cyclic load waveform.

In order to simulate the stress state of important load-bearing structural layers such as subgrades under traffic loads, scholars mostly use sinusoidal waves as dynamic loads to explore the dynamic properties of geotechnical materials. The subgrade soil is characterized by periodic intermittent loading in the actual operation process, and the actual intermittent cyclic load waveform of the subgrade soil is shown in Fig. 1.

The axial static deviatoric stress ${\upsigma }_{\mathrm{s}}$(${\sigma }_{s}={\sigma }_{1}-{\sigma }_{3}$) simulates the static load effect of the superstructure (base layer, rail, ballast, etc.) on the subgrade soil, the alternating load ${\upsigma }_{a}(t)$ simulates the cyclic dynamic load effect when the traffic load passes through, and the confining pressure ${\sigma }_{3}$ simulates the effect of consolidation on the subgrade soil. After the consolidation is completed, it is considered that the specimen has reached a stable state; the axial strain no longer considers the compression deformation of the specimen due to consolidation, and the dynamic stress causing subgrade soil deformation is given by the following equation:

1 $${\upsigma }_{{\text{d}}} \left( t \right) = {\upsigma }_{{\text{s}}} + {\upsigma }_{a} \left( t \right).$$

It should be noted that the static deviatoric stress is the stress that causes the deformation of the subgrade soil, and the magnitude of the static deviatoric stress taken from different tests varies according to the superstructure of the subgrade soil. Furthermore, in the consolidation phase, limited to the test equipment, this paper considers the confining pressure ${\sigma }_{2}={\sigma }_{3}$, the case of ${\sigma }_{2}\ne {\sigma }_{3}$ exclusion.

### Deformation characteristics of subgrade soil under different loads

#### Creep strain analysis of subgrade soil under static deviatoric stress

**Figure 2:**
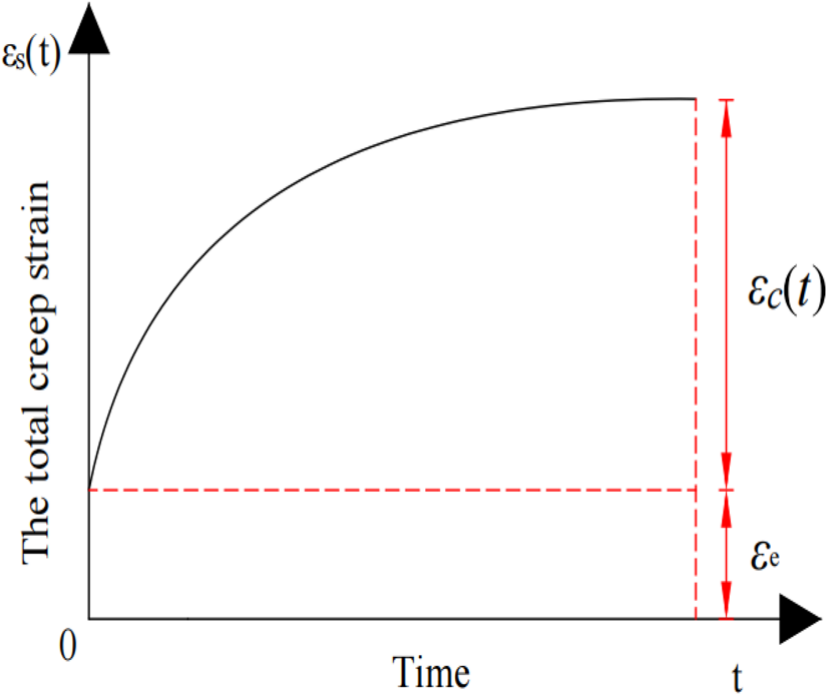
The total creep strain diagram.

Due to the nonlinear creep properties of soil, the total creep strain under static deviatoric stress includes instantaneous strain and creep strain^20^.

2 $${\upvarepsilon }_{{\text{s}}} \left( t \right) = {\upvarepsilon }_{e} + {\upvarepsilon }_{{\text{c}}} \left( t \right),$$

where ${\upvarepsilon }_{e}$ and ${\upvarepsilon }_{\mathrm{c}}(\mathrm{t})$ are the instantaneous strain and creep strain, respectively. The creep strain of soil can be divided into attenuation, stable, and accelerated creep stage^21^. Since the subgrade structural layer has high compaction and stiffness, the static deviatoric stress exerted by the overlying load is far less than the long-term strength of the subgrade structural layer^22^. Therefore, taking no account of the accelerated creep stage in the subgrade soil creep process, the total creep strain of subgrade soil under the action of static deviatoric stress is shown in Fig. 2.

#### Strain response analysis of subgrade soil under intermittent cyclic loading

**Figure 3:**
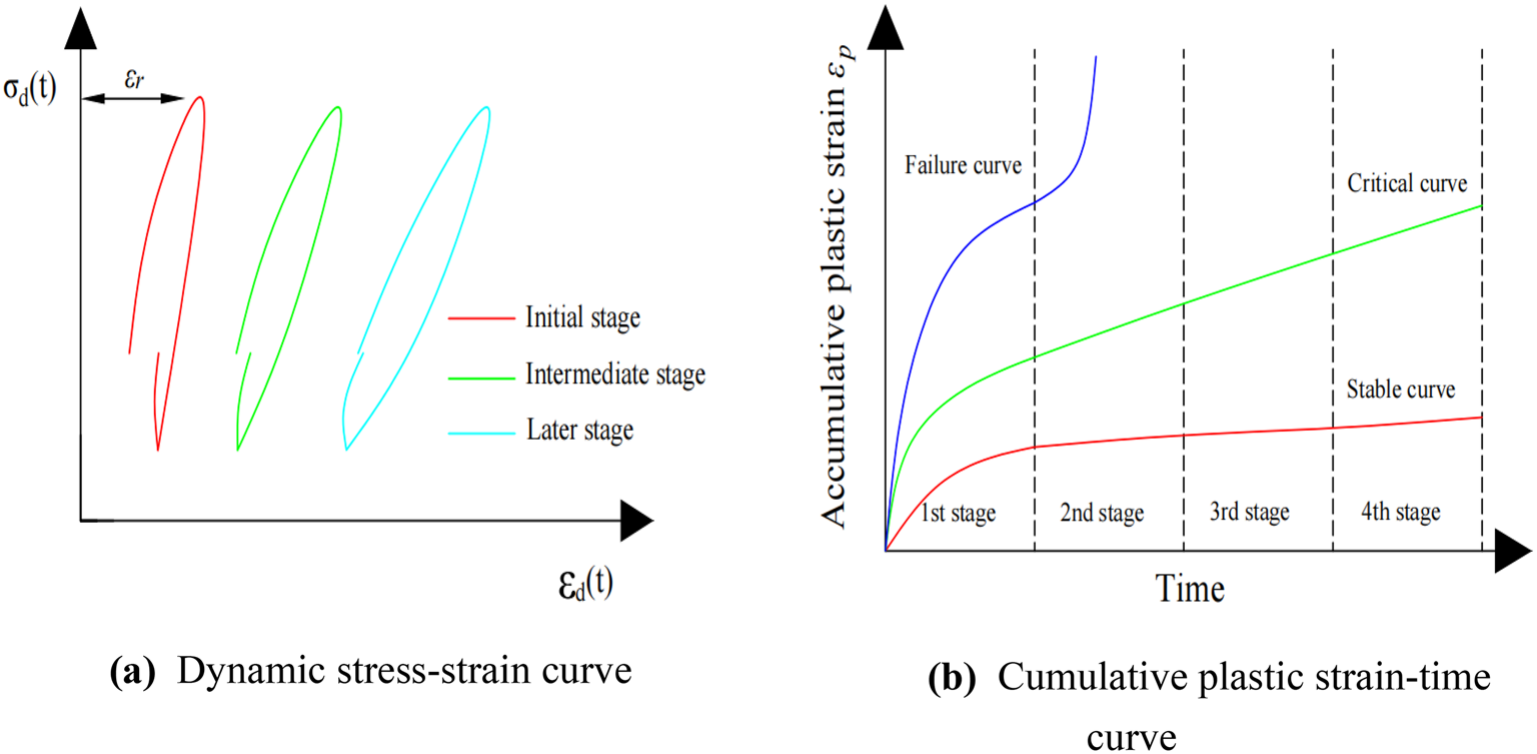
(**a**) Dynamic stress–strain curve. (**b**) Cumulative plastic strain–time curve.

The elastic deformation ${\varepsilon }_{r}$ and plastic deformation ${\varepsilon }_{p}$ of soil will be developed under the action of dynamic load. The hysteretic curve can represent the stress–strain relationship of the soil at various times in a cycle under the action of dynamic load (Fig. 3a). Due to the accumulation of residual plastic deformation, the hysteretic curve is not closed in the initial stage, which indicates that the soil is rapidly compacted and the plastic deformation increases rapidly in the initial stage. With the increase in cyclic vibration time, the soil has been further compacted. The plastic strain generated in one cyclic vibration gradually tends to a small stable value, and the hysteresis curve is almost closed^23^.

The cumulative plastic strain–time curves of soil under intermittent cyclic loading can be divided into three types^24^: (1) stable type; (2) critical type; and (3) failure type (Fig. 3b). The dynamic stress that makes the cumulative plastic strain–time curve evolve into a critical curve is called critical dynamic stress. When the dynamic stress is lower or higher than the critical stress, the cumulative plastic strain–time curve evolves into a stable or failure curve, respectively. Generally, it is onerous to obtain the exact value of the critical dynamic stress of soil under different stress states through laboratory tests^25^, while the failure and stable curves are mainly distributed in the upper left and lower right regions of the figure, and the critical curve is located between the two. Therefore, the critical dynamic stress is considered the median value of this range.

### Total strain of subgrade soil under intermittent cyclic loading

**Figure 4:**
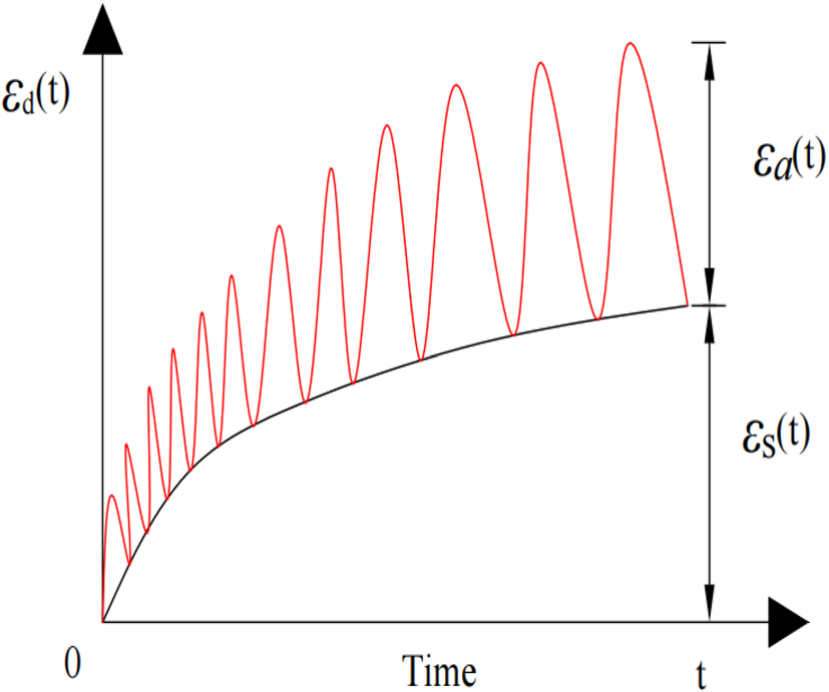
Total dynamic strain–time curve.

Based on the above analysis, it can be seen that the load on the subgrade soil under the intermittent cyclic loading includes static deviatoric stress and alternating stress. When the dynamic stress is less than the critical dynamic stress, the effect of cracks and damage on the deformation of soil specimens is ignored. Consequently, the dynamic strain of subgrade soil under intermittent cyclic loading can be expressed as follows:

3 $$\varepsilon_{{\text{d}}} \left( t \right) = \varepsilon_{{\text{s}}} \left( t \right) + \varepsilon_{a} \left( t \right),$$

where $\varepsilon_{{\text{s}}} \left( t \right)$ and $\varepsilon_{a} \left( t \right)$ indicate the total creep strain induced by deviatoric stress and strain response induced by alternating stress, respectively. The total dynamic strain–time curve of soil under intermittent cyclic loading is shown in Fig. 4.

## Fractional Abel model

### Mathematical preliminaries

#### Definition 1

Let ${L}_{1}={L}_{1}\left(I\right)$ be the class of Lebesgue integral functions on the integral $I=\left(0,+\infty \right)$, and $f\left(t\right)\in {L}_{1}$, $\mathrm{\alpha }\in \left(0,+\infty \right)$, for $t>0$, $\mathcal{R}e\left(\alpha \right)>0$, the Riemann–Liouville integration with the order $\mathrm{\alpha }$ is defined as follows:^26^.

4 $$D_{t}^{{ - {\upalpha }}} f\left( t \right) = \frac{1}{\Gamma \left( \alpha \right)}\mathop \smallint \limits_{0}^{t} \left( {t - \tau } \right)^{\alpha - 1} f\left( t \right)d\tau .$$

The corresponding fractional derivative is defined as

5 $$D_{t}^{{\upalpha }} f\left( t \right) = \frac{{d^{\alpha } }}{{dt^{\alpha } }}\left( {\mathop \smallint \limits_{0}^{t} \left( {t - \tau } \right)^{\sigma - 1} f\left( t \right)d\tau } \right).$$

In the above formula, ${D}_{t}^{-\mathrm{\alpha }}f\left(t\right)$ and ${D}_{t}^{\mathrm{\alpha }}f\left(t\right)$ are the Riemann–Liouville fractional integral operator and derivative operator, respectively. $\mathrm{t}>0$, $\mathrm{\alpha }\ge 0$, n is the smallest positive integer that exceeds $\mathrm{\alpha }$, $\upsigma =\mathrm{n}-\mathrm{\alpha }$, and $\Gamma \left(\alpha \right)$ is the gamma function, $\Gamma \left(\alpha \right)={\int }_{0}^{\infty }{\mathrm{e}}^{-t}{t}^{\mathrm{\alpha }-1}d\uptau$.

It can be seen from Eq. (5) that the Riemann–Liouville integral with order is actually a weighted integral. It is worth noting that, when $\mathrm{\alpha }=1$, the equation will be transformed into a general integral, and the weight is equal to 1; when $\mathrm{\alpha }>1$, the farther the distance between the variable t and the upper limit of the integral, the greater the weight; when $\mathrm{\alpha }<1$, the farther the distance between the variable t and the upper limit of the integral, the smaller the weighted value^27^. This integral structure fully reflects the historical dependence of the system function development^28^. Therefore, the Riemann–Liouville fractional calculus has become a significant means to describe the creep behavior of soil.

### Fractional Abel dashpot

**Figure 5:**
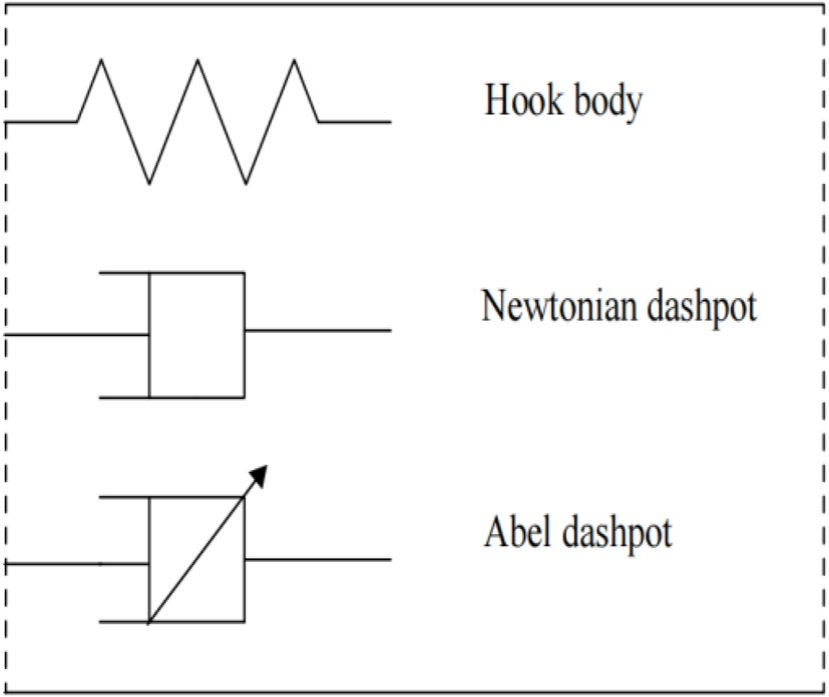
Basic element model.

Soil will undergo elastic, viscoelastic, and plastic deformation during the creep process, and the elastic and plastic deformation of soil can be characterized by a Hooke body and Newtonian dashpot, respectively. As for the viscoelastic deformation of soil, it can be described by a fractional Abel dashpot (Fig. 5).

The constitutive relationship of the Abel dashpot is defined as follows:^29^

6 $${\upsigma }\left( t \right) = {\upeta }^{{\upalpha }} {\text{D}}^{{\upalpha }} {\upvarepsilon }\left( t \right),$$

where $\upeta$ is the viscosity coefficient, $0\le \mathrm{\alpha }\le 1$, and ${\mathrm{D}}^{\mathrm{\alpha }}$ indicates the fractional differentiation.

When $\mathrm{\alpha }=1$, the Abel dashpot in Eq. (6) is a Newtonian dashpot representing an ideal fluid; when $\mathrm{\alpha }=0$, the Abel dashpot becomes a spring, representing an ideal solid; when $0<\mathrm{\alpha }<1$, the Abel dashpot can be considered a comprehensive component used to describe the nonlinear strain processes of geomaterials. Thus, an Abel dashpot can be used to describe the viscoelastic deformation of soil.

**Figure 6:**
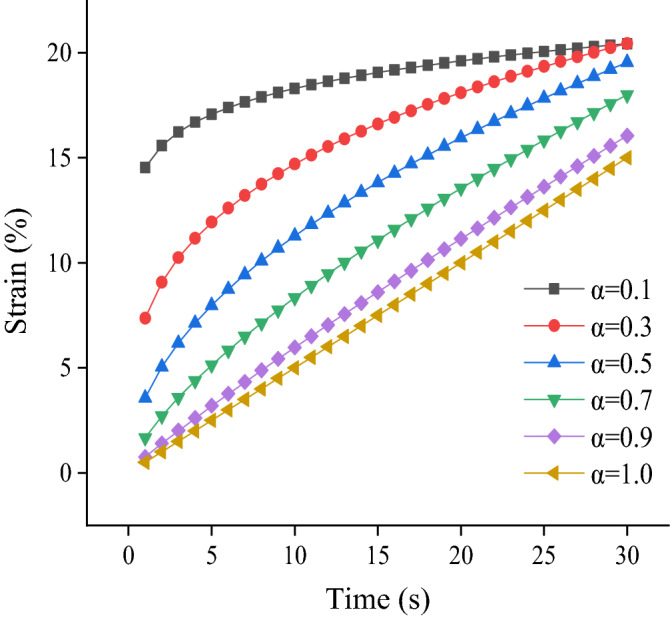
Strain–time curves of Abel dashpot with different orders.

Let $\upsigma (t)={\upsigma }_{\mathrm{s}}$; substituting it into Eq. (6), based on the Riemann–Liouville integral operator, we can obtain the following equation:

7 $${\upvarepsilon }\left( t \right) = \frac{{{\upsigma }_{{\text{s}}} }}{{{\upeta }^{{\upalpha }} }}\frac{{t^{\alpha } }}{{{\Gamma }\left( {1 + {\upalpha }} \right)}},$$

where ${\upsigma }_{\mathrm{s}}$ is the static deviatoric stress; the strain–time curves of different orders are obtained by substituting ${\upsigma }_{\mathrm{s}}=20$ kPa and $\upeta =40\mathrm{ kPa}\cdot {\mathrm{s}}^{\mathrm{\alpha }}$ into Eq. (7) (Fig. 6). When the order tends to 0, the strain–time curve increases nonlinearly, and the strain tends to a certain value, showing the characteristics of an approximate elastic solid—that is, the magnitude of the strain is independent of time. When the order tends to 1, the strain increases linearly with time, establishing the characteristics of an ideal fluid. It can be seen that a change in the order can not only change the magnitude of the strain value, but also the development law of the strain–time curve. Moreover, it should be noted that the indicated coefficient of viscosity in Eq. (7) changes with the change in order. This is because the mechanical state of the soil changes as the order changes, and the corresponding coefficient of viscosity should also change. Therefore, the coefficient of viscosity $\upeta =40\mathrm{ kPa}\cdot {\mathrm{s}}^{\mathrm{\alpha }}$ is assumed here only to evaluate the effect of order change on strain more intuitively.

## Establishment of dynamic constitutive model

### Creep strain of subgrade soil under static deviatoric stress

**Figure 7:**
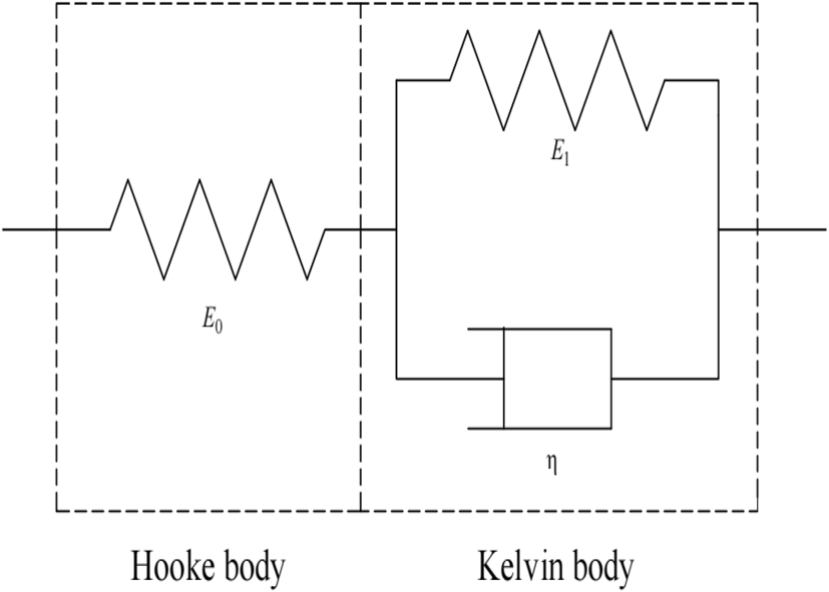
Generalized kelvin model.

According to the viewpoint of geotechnical rheology, the time-dependent deformation of geotechnical materials under long-term loading can be used to quantify the mechanical properties of geotechnical materials by connecting a series of Hooke bodies and Newtonian dashpot in series or in parallel. The generalized Kelvin model consists of a Hooke body and a Kelvin body in series, as shown in Fig. 7.

**Figure 8:**
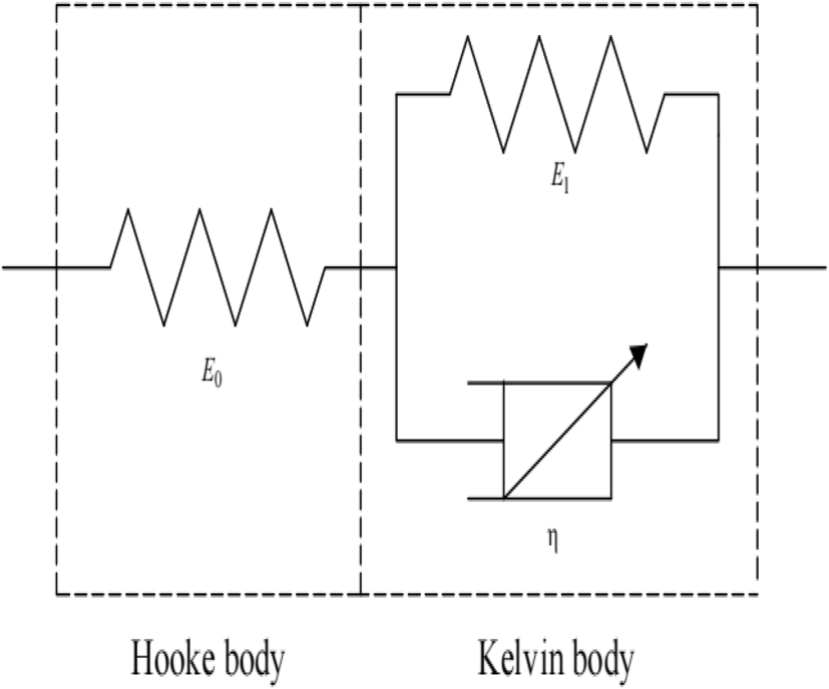
Fractional generalized Kelvin model.

The Hooke body and the Kelvin body can describe the instantaneous strain ${\upvarepsilon }_{e}$ of the soil in the instantaneous stage and the creep strain ${\upvarepsilon }_{\mathrm{c}}(t)$ in the creep stage, respectively. Assuming that the axial deviatoric stress applied to the soil is ${\upsigma }_{{\text{s}}}$, the creep equation of the generalized Kelvin model is

8 $$\varepsilon_{{\text{s}}} \left( t \right) = \frac{{\sigma_{s} }}{{E_{0} }} + \frac{{\sigma_{s} }}{{E_{1} }}\left( {1 - exp\left( { - \frac{{E_{1} }}{{{\upeta }^{\alpha } }}t} \right)} \right),$$

where $\varepsilon_{{\text{s}}} \left( t \right)$ is the creep strain of soil; $E_{0}$ and $E_{1}$ are the elastic modulus in the Hooke and Kelvin body, respectively; and $\upeta$ is the viscosity coefficient. Since the generalized Kelvin model is composed of linear elements, it can only describe the linear creep of geotechnical materials, and is inappropriate for geotechnical materials with complex creep characteristics^30^. Therefore, we replaced the Newtonian dashpot in the Kelvin body with the Abel dashpot, and established a fractional generalized Kelvin model describing the nonlinear creep of the soil (Fig. 8).

The state equation of the fractional generalized Kelvin model is

9 $$\sigma_{s} = E_{0} \varepsilon_{e} = E_{1} \varepsilon_{c} \left( t \right) + \eta^{\alpha } D^{\alpha } \left[ {\varepsilon_{c} \left( t \right)} \right],$$

where ${\varepsilon }_{e}$ is the instantaneous strain and ${\varepsilon }_{c}(t)$ is the creep strain of the soil at time t. For fractional generalized Kelvin bodies, the corresponding state equation is

10 $$\sigma_{s} = E_{1} \varepsilon_{c} \left( t \right) + \eta^{\alpha } D^{\alpha } \left[ {\varepsilon_{c} \left( t \right)} \right].$$

Substituting ${\upsigma }_{\mathrm{s}}={\upsigma }_{\mathrm{s}}\uptheta (\mathrm{t})$ ($\theta (\mathrm{t})$ is the Heaviside step function) into Eq. (10), and then conducting a Laplace transform, we obtain the following equation:

11 $$\varepsilon_{c} \left( s \right) = \frac{b}{{s\left( {a + s^{\alpha } } \right)}},$$

where $\frac{{E}_{1}}{{\eta }^{\alpha }}=a$, $\frac{{\sigma }_{s}}{{\eta }^{\alpha }}=b$. By performing an inverse Laplace transform on Eq. (11), we obtain

12 $$\varepsilon_{c} \left( t \right) = bt^{\alpha } E_{\alpha ,\alpha + 1}^{\left( 0 \right)} \left( { - at^{\alpha } } \right),$$

where

13 $$E_{{{\upalpha },{\upbeta }}}^{{\text{(k)}}} \left( z \right) = \mathop \sum \limits_{j = 0}^{\infty } \frac{{\left( {j + k} \right)!z^{j} }}{{j!\Gamma \left( {\alpha \left( {j + k} \right) + \beta } \right)}} \quad (\alpha > 0, \beta > 0).$$

${\text{E}}_{{{\upalpha },{\upbeta }}}^{{\text{(k)}}} \left( {\text{z}} \right)$ is the Mittag–Leffler function with two parameters. Substituting Eq. (13), $a$ and $b$ into Eq. (12) allows us to obtain creep strain:

14 $$\varepsilon_{c} \left( t \right) = \frac{{\sigma_{s} }}{{\eta^{\alpha } }}\mathop \sum \limits_{k = 0}^{\infty } \frac{{\left( { - E_{1} /{\upeta }^{{\upalpha }} } \right)^{k} t^{{\alpha \left( {k + 1} \right)}} }}{{\Gamma \left( {\alpha k + \alpha + 1} \right)}}.$$

Considering the strain of the Hooke body and Kelvin body in the fractional generalized Kelvin model, the creep constitutive equation of the fractional generalized Kelvin model can be obtained as follows:

15 $$\varepsilon_{s} \left( t \right) = \frac{{\sigma_{s} }}{{E_{0} }} + \frac{{\sigma_{s} }}{{\eta^{\alpha } }}\mathop \sum \limits_{k = 0}^{\infty } \frac{{\left( { - E_{1} /{\upeta }^{{\upalpha }} } \right)^{k} t^{{\alpha \left( {k + 1} \right)}} }}{{\Gamma \left( {\alpha k + \alpha + 1} \right)}}.$$

### Strain response of subgrade soil under intermittent cyclic loading

#### Strain response of fractional generalized Kelvin model under continuous cyclic loading

##### The dynamic stress amplitude is less than or equal to the critical dynamic stress, i.e., ${{\varvec{\sigma}}}_{\mathbf{d}}\le {{\varvec{\sigma}}}_{{\varvec{c}}{\varvec{r}}}$

When soil is subjected to alternating loads in the loading stage, the generated dynamic strain lags behind the dynamic stress, leading to the emergence of a strain hysteresis phase difference. The strain hysteresis phase differences of elastic solids and ideal viscous fluids are 0 and $\uppi /2$, respectively. Soil commonly exhibits the properties of both elastic solids and ideal viscous fluids in the meantime, i.e., viscoelasticity. Assuming that the strain hysteresis phase difference of the soil is $\theta$, $0<\theta <\uppi /2$, and the hysteresis time is $\theta /\upomega$, $\upomega$ is the angular velocity. Under the action of alternating load, the oscillating stress on the soil is assumed to be

16 $$\sigma_{a} \left( t \right) = \sigma_{d} sin\left( {wt} \right) = \sigma_{d} e^{iwt} ,$$

where ${\sigma }_{d}$ (as shown in Fig. 1) is the amplitude of the oscillatory stress, *i* is the imaginary unit, $w=2\pi f$, and $f$ is the frequency of alternating load. On the basis of the viscoelasticity theory, the strain response of soil in one cycle period under steady state condition is

17 $$\varepsilon_{a} \left( t \right) = \varepsilon_{1} sin\left( {wt + \theta } \right) = \varepsilon_{1} e^{{i\left( {wt + \theta } \right)}} = \varepsilon^{*} e^{iwt} ,$$

where $\varepsilon_{1}$ is the amplitude of the alternating strain, $\varepsilon^{*}$ is the complex strain, and $\varepsilon_{1}$ and $\theta$ are calculated as follows:

18 $$\varepsilon_{1} = \sigma_{d} \sqrt {J_{1}^{2} + J_{2}^{2} }$$

19 $$\theta = {\text{arctan}}\left( {J_{2} /J_{1} } \right),$$

where ${J}_{1}$ and ${J}_{2}$ are the energy storage compliance and loss compliance, respectively.

1. Hooke body

Based on the above analysis, the state equation of the Hooke body under alternating stress ${\sigma }_{\mathrm{d}}{e}^{iwt}$ is

20 $$\sigma_{{\text{d}}} e^{iwt} = E_{0} \cdot \varepsilon^{*} e^{iwt} .$$

$E_{0}$ is the elastic modulus of the Hooke body. When Eq. (20) is written in the form of complex compliance, we can obtain

21 $${\text{J}}^{*} \left( {{\text{iw}}} \right) = \frac{{\varepsilon^{*} }}{{\sigma_{d} }} = \frac{1}{E} = J_{H1} - iJ_{H2} .$$

The storage compliance of the Hooke body is ${J}_{H1}=1/E$ and the loss compliance is ${J}_{H2}=0$.

(2) Fractional Abel dashpot

For a fractional Abel dashpot, the state equation is

22 $$\sigma_{d} e^{iwt} = \eta^{\alpha } \left( {iw} \right)^{\alpha } \varepsilon^{*} e^{iwt} .$$

Similarly, Eq. (22) can be written in the form of complex compliance:

23 $${\text{J}}^{*} \left( {{\text{iw}}} \right) = \frac{{\varepsilon^{*} }}{{\sigma_{d} }} = \frac{1}{{\eta^{\alpha } \left( {iw} \right)^{\alpha } }}.$$

Due to $\left( i \right)^{\alpha } = {\text{cos}}\left( {\frac{\alpha \pi }{2}} \right) + i \cdot sin\left\{ {\left. {\frac{\alpha \pi }{2}} \right)} \right.$, Eq. (23) can be written as follows:

24 $$\frac{{\varepsilon^{*} }}{{\sigma_{d} }} = \frac{{{\text{cos}}\left( {\frac{\alpha \pi }{2}} \right)}}{{\left( {\eta w} \right)^{\alpha } }} - i\frac{{{\text{sin}}\left( {\frac{\alpha \pi }{2}} \right)}}{{\left( {\eta w} \right)^{\alpha } }} = J_{A1} - iJ_{A2} .$$

Correspondingly, the energy storage compliance of the fractional Abel dashpot is ${J}_{A1}=\frac{\mathrm{cos}(\frac{\alpha \pi }{2})}{{(\eta w)}^{\alpha }}$ and the loss compliance is ${J}_{A2}=\frac{\mathrm{sin}(\frac{\alpha \pi }{2})}{{(\eta w)}^{\alpha }}$.

(3) Fractional Kelvin body

For the fractional Kelvin body, the state equation is

25 $$\sigma_{d} e^{iwt} = E_{1} \cdot \varepsilon^{*} e^{iwt} + \eta^{\alpha } \left( {iw} \right)^{\alpha } \varepsilon^{*} e^{iwt} ,$$

where ${E}_{1}$ is the elastic modulus of the Hooke body in the fractional Kelvin body. Rewriting Eq. (25) into the form of complex compliance, we obtain

26 $${\text{J}}^{*} \left( {iw} \right) = \frac{{\varepsilon^{*} }}{{\sigma_{d} }} = \frac{1}{{E_{1} + \left( {\eta w} \right)^{\alpha } i^{\alpha } }} = \frac{1}{{E_{1} + \left( {\eta w} \right)^{\alpha } cos\left( {\frac{\alpha \pi }{2}} \right) + i \cdot \left( {\eta w} \right)^{\alpha } sin\left( {\frac{\alpha \pi }{2}} \right)}}.$$

Assuming $c={E}_{1}+{(\eta w)}^{\alpha }cos(\frac{\alpha \pi }{2})$, $d={(\eta w)}^{\alpha }sin(\frac{\alpha \pi }{2})$, Eq. (26) can be rewritten as follows:

27 $${\text{J}}^{*} \left( {iw} \right) = \frac{{\varepsilon^{*} }}{{\sigma_{d} }} = \frac{1}{c + d \cdot i} = \frac{c}{{c^{2} + d^{2} }} - i\frac{d}{{c^{2} + d^{2} }} = J_{k1} - iJ_{k2} .$$

The energy storage compliance of the fractional Kelvin body is ${J}_{k1}=\frac{c}{{c}^{2}+{d}^{2}}$, and the loss compliance is ${J}_{k2}=\frac{d}{{c}^{2}+{d}^{2}}$.

Synthetically, the energy storage compliance and loss compliance of the fractional generalized Kelvin model under continuous cyclic loading are

28 $$J_{1} = J_{H1} + J_{k1} = \frac{1}{{E_{0} }} + \frac{c}{{c^{2} + d^{2} }}$$

29 $$J_{2} = J_{H2} + J_{k2} = \frac{d}{{c^{2} + d^{2} }}.$$

From Eqs. (17)–(19), (28), and (29), it can be found that, under the condition of continuous cyclic loading, when the dynamic stress amplitude is less than or equal to the critical dynamic stress, the dynamic strain response of the fractional generalized Kelvin model is

30 $$\varepsilon_{a} \left( t \right) = \sigma_{d} \sqrt {J_{1}^{2} + J_{2}^{2} } sin\left( {wt + {\text{arctan}}\left( {J_{2} /J_{1} } \right)} \right).$$

Furthermore, considering that the strain response of soil caused by dynamic stress is not only related to the dynamic stress amplitude, but also affected by the confining pressure, which is mainly manifested as the same dynamic stress amplitude, the larger the confining pressure, the smaller the strain response caused. Thus, the dynamic stress amplitude in Eq. (30) is replaced by the cyclic stress ratio ($\mathrm{CSR}={\sigma }_{d}/2{\sigma }_{3}$), and the dynamic response strain expression of the fractional generalized Kelvin model, considering the effect of confining pressure and dynamic stress amplitude level, is obtained:

31 $$\varepsilon_{a} \left( t \right) = \frac{{\sigma_{d} }}{{2\sigma_{3} }}\sqrt {J_{1}^{2} + J_{2}^{2} } sin\left( {wt + {\text{arctan}}\left( {J_{2} /J_{1} } \right)} \right).$$

##### The dynamic stress amplitude is larger than the critical dynamic stress, i.e., ${{\varvec{\sigma}}}_{\mathbf{d}}>{{\varvec{\sigma}}}_{{\varvec{c}}{\varvec{r}}}$

**Figure 9:**
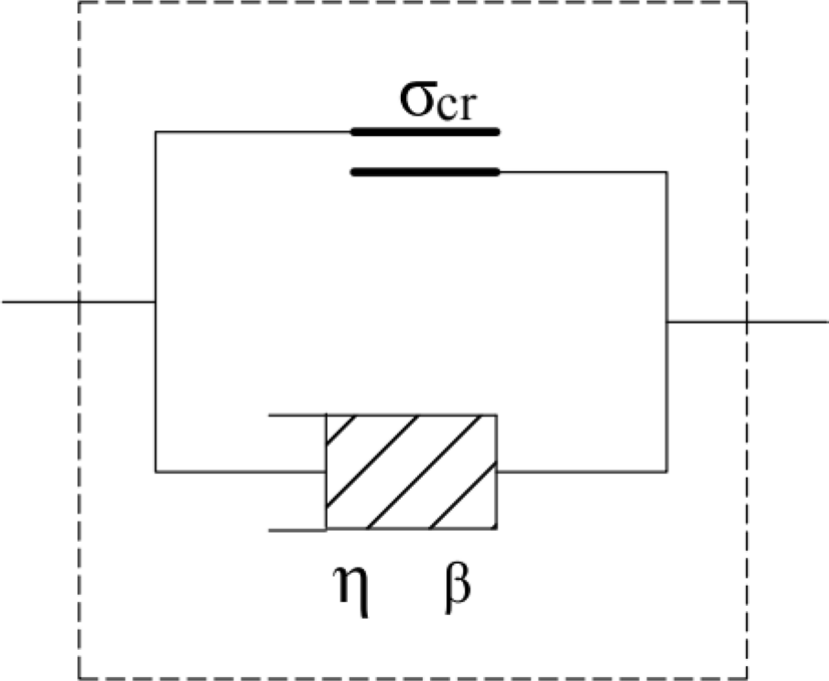
Nonlinear viscoplastic body model considering damage.

In previous studies, scholars have proposed different damage criteria based on different research objects and actual engineering control values. Therefore, the current damage criteria of soil under cyclic loading are not uniform. However, regardless of the damage criteria, from a microscopic analysis, when the amplitude of dynamic stress is slightly larger than the critical dynamic stress, the soil particles start to misalign and move, the interparticle connection is partially destroyed, the cumulative plastic strain–time curve shows an obvious turning point, and the soil will gradually reach the damage criterion over time; when the amplitude of dynamic stress is much larger than the critical dynamic stress, the connection between soil particles will break and misalign, and the internal connection of the soil will become more loose. When the amplitude of dynamic stress is much higher than the critical dynamic stress, the connection between soil particles will be broken and misaligned, the internal connection of soil body will be more loosened and partly out of contact, the stability will be greatly reduced, and the plastic strain will increase sharply, showing obvious acceleration characteristics^31^. Therefore, when the dynamic stress amplitude is greater than the critical dynamic stress, the soil is no longer regarded as a viscoelastic material; hence, its strain response is not derived in the paper. Besides, the fractional order generalized Kelvin model cannot fully reflect the acceleration characteristics of soil in the failure stage. Combined with the development characteristics of the dynamic strain–time failure curve, we proposed a nonlinear viscoplastic model considering damage (Fig. 9) to quantitatively describe the accumulated plastic strain of the subgrade soil and characterize the strain pattern of the soil during the damage phase. The expression is as follows:

32 $$\varepsilon_{{\text{d}}} \left( t \right) = \frac{{H(\sigma_{d} - \sigma_{cr} )}}{{{\eta e}^{{ - {\beta t}}} }}t,$$

where $\upeta$ is the viscosity coefficient and $\upbeta$ is defined as a parameter related to the soil property, which reflects the reflect the speed of reduction of soil viscosity coefficient. ${H(\sigma }_{d}-{\sigma }_{cr})$ is given as follows:

33 $$H(\sigma_{d} - \sigma_{cr} ) = \left\{ {\begin{array}{*{20}l} 0 \hfill & {(\sigma_{d} \le \sigma_{cr} )} \hfill \\ {\sigma_{d} - \sigma_{cr} } \hfill & {(\sigma_{d} > \sigma_{cr} )} \hfill \\ \end{array} } \right..$$

**Figure 10:**
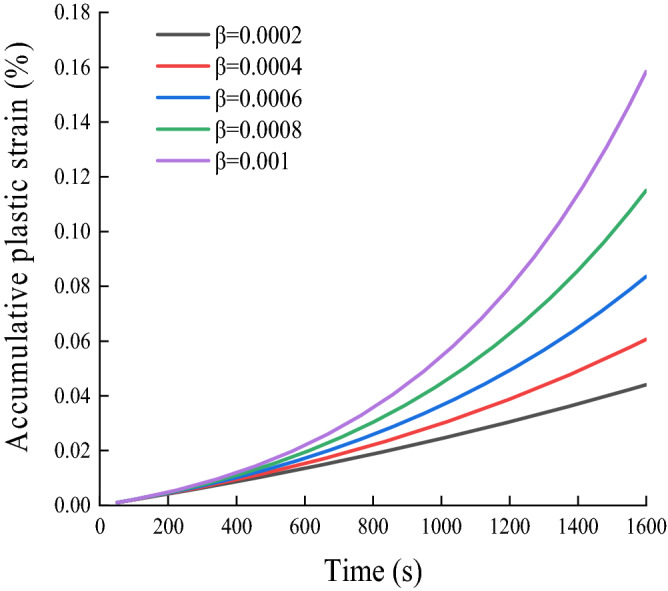
Development curve of cumulative plastic strain of nonlinear viscoplastic model considering damage.

From Eqs. (32) and (33), it can be seen that the model takes into account the dynamic stress amplitude, critical dynamic stress magnitude, and the joint effect of the viscosity coefficient on the cumulative plastic strain of the soil, so as to evaluate the development law of cumulative plastic strain of soil as comprehensively and accurately as possible. Assuming ${H(\sigma }_{d}-{\sigma }_{cr})=20 \mathrm{kPa}$, $\upeta =10\mathrm{ kPa}\cdot \mathrm{s}$, the cumulative plastic strain development curve of the nonlinear viscoplastic model considering damage under different values can be obtained (Fig. 10). It can be seen that, with the increase in parameter $\upbeta$, the development of the cumulative plastic strain demonstrates an obvious acceleration trend, which can reflect the acceleration characteristics of the soil in the failure stage.

#### Strain response of fractional generalized Kelvin model under intermittent cyclic loading

Based on the intermittent cyclic load waveform shown in Fig. 1 and the dynamic response strain of the fractional generalized Kelvin model under continuous cyclic loading given by Eq. (31), when the dynamic stress amplitude is less than or equal to the critical dynamic stress, the dynamic response strain of the fractional generalized Kelvin model under the action of intermittent cyclic loading is

34 $$\varepsilon_{a} \left( t \right) = \left\{ {\begin{array}{*{20}l} {\frac{{\sigma_{d} }}{{2\sigma_{3} }}\sqrt {J_{1}^{2} + J_{2}^{2} } sin\left( {wt + arctan\left( {J_{2} /J_{1} } \right)} \right)} \hfill & {\left( {Loading\, stage} \right)} \hfill \\ 0 \hfill & {\left( {Intermittent\, stage} \right)} \hfill \\ \end{array} } \right..$$

When the dynamic stress amplitude is greater than the critical dynamic stress, the intermittent stage does not reduce the development level of the plastic strain in the next stage, and the cumulative plastic strain of the soil maintains rapid growth^18^. Consequently, the strain response during the intermittent stage is consistent with the loading stage. When the dynamic stress amplitude is greater than the critical dynamic stress, the cumulative plastic strain equation of soil under intermittent cyclic loading is expressed as follows:

35 $$\varepsilon_{{\text{d}}} \left( t \right) = \frac{{H(\sigma_{d} - \sigma_{cr} )}}{{{\eta e}^{{ - {\beta t}}} }}t.$$

### Deformation constitutive equation of subgrade soil under intermittent cyclic loading

**Figure 11:**
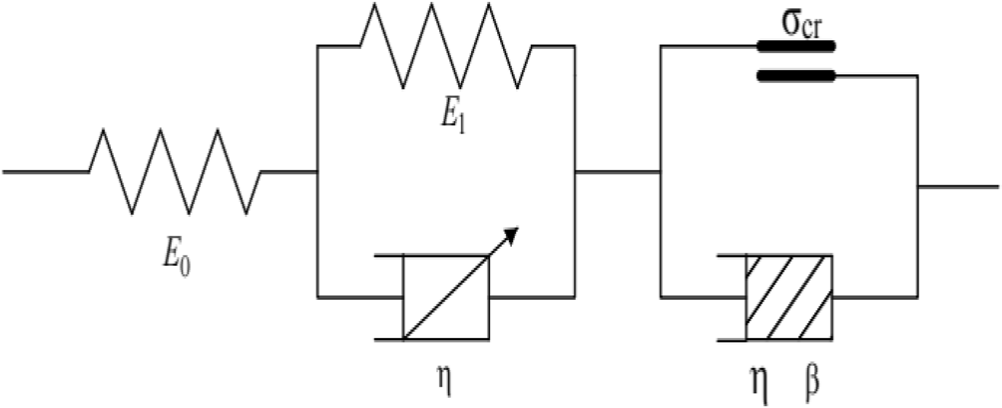
Deformation constitutive model of soil under intermittent cyclic loading.

For the purpose of accurately assessing the cumulative plastic strain development of subgrade soil, the soil described in this paper is not in the worst state. In practice, most subgrade soil has a high compaction degree and low permeability, and vehicles such as trains, cars, and airplanes on the subgrade soil pass over faster, which leads to an inability to discharge the moisture in the subgrade soil during the loading stage. When vehicles such as trains pass over, the excess pore water pressure caused by them begins to dissipate^32^. Therefore, during the dynamic triaxial test, the drain valve needs to be opened for drainage during the intermittent stage. Additionally, under the condition of drainage, the cumulative plastic strain of the soil remains basically unchanged in the intermittent stage^33^. Accordingly, the constitutive model does not embody the deformation constitutive model expression of the soil in the intermittent stage. In accordance with Eqs. (3), (15), (34), and (35), the deformation constitutive model and expressions of subgrade soil under intermittent cyclic loading are shown in Fig. 11 and Eqs. (36) and (37), respectively.

1. $$\sigma_{d} \le \sigma_{cr}$$
  36 $$\varepsilon_{{\text{d}}} \left( t \right) = \frac{{\sigma_{s} }}{{E_{0} }} + \frac{{\sigma_{s} }}{{\eta^{\alpha } }}\mathop \sum \limits_{k = 0}^{\infty } \frac{{\left( { - E_{1} /{\upeta }^{{\upalpha }} } \right)^{k} t^{{\alpha \left( {k + 1} \right)}} }}{{\Gamma \left( {\alpha k + \alpha + 1} \right)}} + \frac{{\sigma_{d} }}{{2\sigma_{3} }}\sqrt {J_{1}^{2} + J_{2}^{2} } sin\left( {wt + {\text{arctan}}\left( {J_{2} /J_{1} } \right)} \right){ }$$
2. $$\sigma_{d} > \sigma_{cr}$$
  37 $$\varepsilon_{{\text{d}}} \left( t \right) = \frac{{H(\sigma_{d} - \sigma_{cr} )}}{{{\eta e}^{{ - {\beta t}}} }}t$$

## Applicability verification of the model and analysis of fitting parameters

The validity and applicability of the model are discussed through the cumulative strain test results of the fine-grained soil^18^ and saturated soft clay^33^ under intermittent loading; the parameters $\alpha$ and $\eta$ in the dynamic constitutive model are also analyzed. During the intermittent cycle loading test, considering the high compaction of the subgrade soil and the low permeability coefficient, it is difficult to drain the water in the subgrade soil during the short time when a train passes. Therefore, during the loading phase, the drainage valve is closed and the specimen is not drained. When the train leaves, the pore pressure gradually dissipates. In order to simulate the process of pore pressure dissipation, the specimen is drained by opening the drainage valve in the interval phase. The number of loading and interval stages is set not only considering the time cost and the limitation of the apparatus itself, but also the transportation condition of the upper part of the actual subgrade soil and the damage criteria of the subgrade soil. Furthermore, in order to explore the deformation law of the subgrade soil under different stress paths, the dynamic stress amplitude of different loading stages can be varied. Due to space limitations, the test equipment, specimen size, test methods, and other information are not listed. The basic physical and mechanical properties of fine-grained soil and soft clay are shown in Tables 1 and 2, respectively.

**Table 1:** Basic physical and mechanical properties of fine-grained soil.

Gravity ${G}_{s}$	Maximum dry density $\rho_{ \cdot d \cdot max} \left( {{\text{g}} \cdot {\text{cm}}^{3} } \right)$	Optimum moisture content ${w}_{opt} (\%)$	In-site moisture content ${w}_{ins} (\%)$	Liquid limit ${w}_{\mathrm{L}} (\%)$	Plastic limit ${w}_{\mathrm{p}} (\%)$	Plastic index ${I}_{p}$
2.71	1.96	11.8	15.0	26.0	18.2	7.8

**Table 2:** Basic physical and mechanical properties of soft clay.

Gravity ${G}_{s}$	Natural moisture content *w* (%)	Initial void ratio ${e}_{0}$	Liquid limit ${w}_{\mathrm{L}} (\%)$	Plastic limit ${w}_{\mathrm{p}} (\%)$	Compression index	Illite (%)	Clay content (%)	Silt content (%)
2.66	62.5	1.75	66	28	0.539	61.3	57	41

### Applicability verification of the model

**Figure 12:**
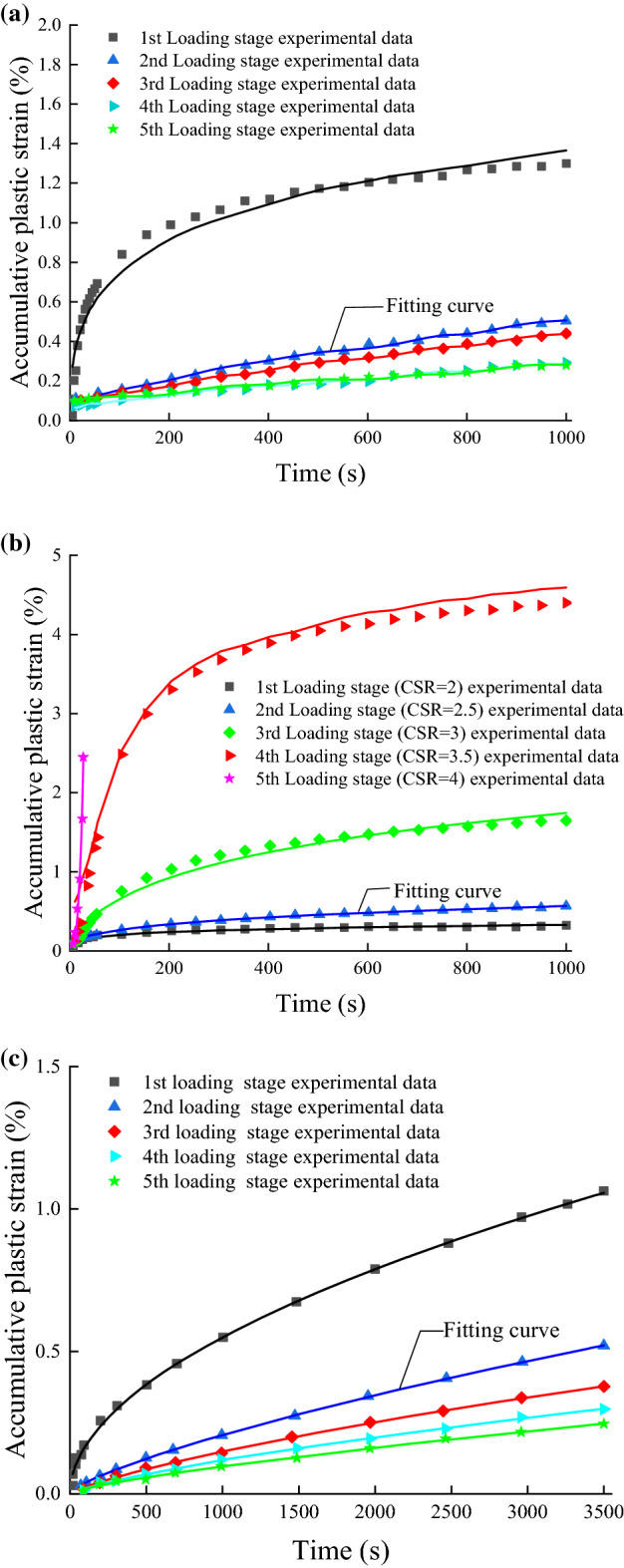
(**a**) Comparison between experimental data and model fitting curve of fine-grained soil under single-stage intermittent cyclic loading (${w}_{opt}=11.8$%, ${\sigma }_{3}=60$ kPa, CSR = 2). (**b**) Comparison between experimental data and model fitting curve of fine-grained soil under multi-stage intermittent cyclic loading (${w}_{opt}=11.8\%$, ${\sigma }_{3}=60$ kPa, CSR = 2–2.5–3–3.5–4). (**c**) Comparison between experimental data and model fitting curve of saturated soft clay under single-stage intermittent cyclic loading (${\sigma }_{3}=100$ kPa, CSR = 0.15).

The validity of the dynamic constitutive model depends on its ability to fully fit the experimental data. 1stOpt mathematical optimization software is used to verify the applicability of the model; based on the simulated annealing algorithm (SAA), the dynamic triaxial experimental data for various soils and loading methods was used to fit the constitutive model to explore the applicability of the model. The fitting effect is shown in Fig. 12.

Comparing Fig. 12a,b, it can be seen that the model agrees well with the dynamic triaxial test data of fine-grained soil under single-stage and multistage intermittent cyclic loading, which indicates that the model can well predict the cumulative plastic strain of soil under single-stage and multistage intermittent cyclic loading. The reason is that we considered the coupled action of confining pressure and dynamic stress amplitude in the strain response; i.e., the cyclic stress ratio (CSR) is used to replace the dynamic stress amplitude to reduce the overestimated cumulative plastic strain due to dynamic stress, while endowing the model with more flexibility in application. Moreover, it is observed from Eq. (36) that, although the expression of the constitutive equation is complex, it only contains four parameters to be fitted, which reduces the uncertainty of the model to some extent and increases the fitting accuracy. Meanwhile, the model also considers the combined effects of static deviatoric stress, vibration frequency, and cyclic stress ratio on the cumulative plastic strain of the soil. The introduction of these influencing factors aims to accurately evaluate the development of the cumulative plastic strain of the soil. Therefore, the relevant staff can make corresponding preparations during the construction and operation of the structures to ensure the normal operation of the traffic facilities on subgrade.

It can be seen from Fig. 12a,c that the model can also well describe the cumulative plastic strain–time curves of fine-grained soil and saturated soft clay. Although the various soil types will lead to different initial strain rates under the same dynamic stress, on the whole, when the dynamic stress is less than the critical dynamic stress, the cumulative plastic strain of the soil rapidly increases in the initial stage, and then the strain rate decays and tends to 0. The model has a favorable effect on the stable curves of the two soils, which means the constitutive model can describe the stable curves of different soil samples. When the dynamic stress is greater than the critical dynamic stress, the cumulative plastic strain–time curve of the soil will develop into a failure-type curve. It can be seen from the leftmost curve in Fig. 12b that, in the initial stage of strain, i.e., the lower half of the curve, the nonlinear viscoplastic model, considering damage, can reflect the nonlinear increase in strain. In the accelerating stage, the upper part of the curve, the model fits the experimental data well, which means that the nonlinear viscoplastic model can not only reflect the nonlinear increase in the cumulative plastic strain of the soil, but also the accelerated characteristics of the soil failure. To sum up, the deformation constitutive model can not only characterize the development law of three kinds of soil cumulative plastic strains (stable type, critical type, and failure type), but also the combined effect of confining pressure, frequency, and dynamic stress amplitude levels on the cumulative plastic strain of soil.

### Analysis of model fitting parameters

**Figure 13:**
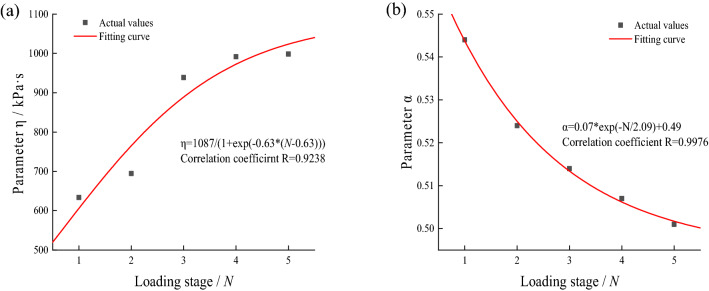
Fitting parameters of fine-grained soil under single-stage intermittent loading and its variation curve with loading stage.

**Figure 14:**
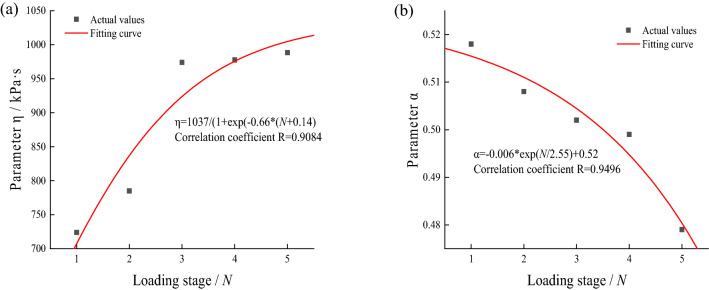
Fitting parameters of saturated soft clay under single-stage intermittent loading and its variation curve with loading stage.

Through considerable experimental results, Li et al. pointed out that the fitting parameters in the fitting equation will change with changes in stress conditions, soil types, and physical properties^34^. Apparently, under the action of intermittent cyclic loading, the soil will be affected by the stress history in different loading stages and show different mechanical properties, which leads to continuous changes in the subsequent fitting parameters. Based on this, we analyze the variation of parameters $\alpha$ and $\eta$ with the increase in loading stage *N*. Since the parameters ${E}_{0}$ and ${E}_{1}$ in the model cannot reflect the mechanical state of the soil, they are not discussed. The variation law of parameters $\alpha$ and $\eta$ of fine-grained soil and saturated soft clay under single-stage intermittent cyclic loading with the loading stage *N* are shown in Figs. 13 and 14.

It can be seen that the fitting parameters $\alpha$ and $\eta$ tend to decay and rise, respectively, with the increase in loading stage *N*. The reason is that the existence of the loading intermittent stage and the drainage effect will eliminate the accumulated pore water pressure in the loading stage, and the internal particles and structure of the soil will be adjusted. The distance between them decreases, the resistance to particle movement and deformation increases, the strain rate of the soil decays, and the viscosity coefficient rises. Besides, since the existence of the intermittent stage can be regarded as the reconsolidation process of the soil specimen^16^, when the dynamic stress is less than the critical dynamic stress, the specimen gradually becomes denser with the increase in intermittent time, so its mechanical property gradually tends to an ideal solid. Consequently, the parameter $\alpha$ gradually decays with increasing loading stages. The empirical formula and fitting effect of parameters $\alpha$ and $\eta$ with the loading stage *N* are given in the legend.

### Model comparison

To date, many dynamic constitutive models for soil have been proposed by scholars; these models were improved to satisfy various situations, such as the exponential model initially proposed by Monismith, the hyperbolic model, the improved exponential model considering the effect of stress level, and the improved hyperbolic model. On the whole, the fitting effect of the models to the accumulative strain curve is better and better; the equations of the models above are given in Eqs. (38)–(41):

Exponential model:^35^

38 $${\upvarepsilon }_{{\text{d}}} \left( {\text{t}} \right) = aN^{b} ;$$

Hyperbolic model:^36^

39 $${\upvarepsilon }_{{\text{d}}} \left( {\text{t}} \right) = \frac{N}{a + bN};$$

Improved exponential model:^34^

40 $${\upvarepsilon }_{{\text{d}}} \left( {\text{t}} \right) = a\left( {\frac{{{\upsigma }_{{\text{d}}} }}{{{\upsigma }_{{\text{s}}} }}} \right)^{{\text{m}}} N^{b} ;$$

Improved hyperbolic model:^37^

41 $${\upvarepsilon }_{{\text{d}}} \left( {\text{t}} \right) = d\left( {{\updelta }^{N} - 1} \right) + \frac{{bN^{m} }}{{1 + cN^{m} }},$$

where *N* is the circulation times, and $\mathrm{t}=NT$; *T* is the period of cyclic load; and *a*, *b*, *c*, and* m* are fitting parameters. $\updelta$ is related to the state of soil, while *d* is the form factor of accumulative strain.

For the exponential model, the cumulative plastic strain of the specimen increases infinitely with the vibration time, which is contrary to the characteristic that the deformation of the stable specimen tends to be stable eventually. For the hyperbolic model, when the vibration time is close to infinity, the cumulative plastic strain of the soil will eventually tend to a certain value, $1/b$, which is not consistent with the development law of the cumulative plastic strain of the failure specimen. For the purpose of quantifying the effect of stress level on the cumulative plastic strain of soil, Li et al. added the ratio of dynamic stress amplitude to the static deviatoric stress in the coefficients of the exponential model. Considering the respective strengths and shortages of the exponential model and the hyperbolic model, Zang et al. made appropriate improvements to the exponential and hyperbolic models, and combined them to satisfy the characteristics of both stable and failure curves.

**Figure 15:**
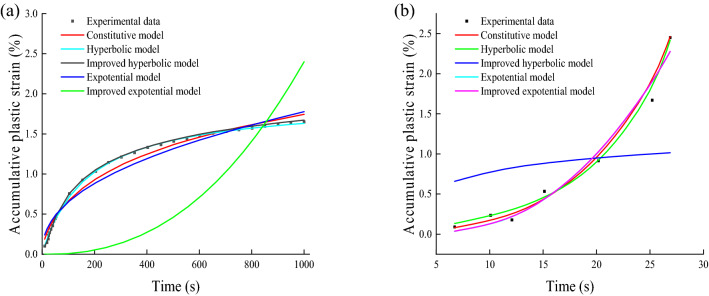
Comparison of fitting effects of different models: (**a**) CSR = 3; (**b**) CSR = 4.

Nevertheless, the above models are all empirical models that can only be applied to a certain type of soil or a specific situation. In order to fine-tune the fitting capacity of each model, Matlab 2017 software was used to fit the above models to the experimental data of a cyclic stress ratio (CSR) of 3 and 4 for fine-grained soil under multistage loading, respectively. The fitting results were obtained as shown in Fig. 15.

It can be seen from Fig. 15 that, with the increase in CSR, the cumulative plastic strain–time curve evolves from a stable curve to a failure curve. Therefore, the fitting effect of the above empirical model on the data also changes correspondingly. The specific result is that the improved exponential model has a poor fitting effect on the stable curve, but can describe the characteristics of the failure curve well. On the contrary, although the improved hyperbolic model has a desirable effect on the fitting of the stable curve, the fitting effect on the destructive curve is undesirable. It can be seen from the comparison that the deformation constitutive model can well capture the characteristics of stable and failure curves. Additionally, although these empirical models have certain fitting capabilities, the physical meanings of the fitting parameters in most models are not clear and cannot reflect the mechanical properties of the soil. In contrast, the deformation constitutive model includes the joint action of various influencing factors such as confining pressure, frequency, and dynamic stress amplitude on the cumulative plastic strain of the soil, and parameters $\alpha$ and $\eta$ can reflect the mechanical properties of the soil.

It is observed that the frequency, dynamic stress amplitude, and other parameters in the deformation constitutive model can be changed according to the actual traffic load. Therefore, in practical engineering applications, the deformation constitutive model can be used for monitoring the subgrade deformation of transport lines with increased axle weight or new trains in the early operation. For the prediction of subgrade settlement of old railroad transport lines, the empirical model can be used.

## Conclusions

In accordance with the characteristics that subgrade soil will be affected by the intermittent cyclic load generated by the vehicle and the static load generated by the superstructure, the cumulative plastic strain of the subgrade soil is divided into creep strain caused by static deviatoric stress and strain response caused by alternating load. By means of the fractional calculus theory, generalized Kelvin model, and viscoplastic theory, the deformation constitutive model of soil under intermittent cyclic loading is established.

Using the experimental data of fine-grained soil and saturated soft clay under intermittent cyclic loading, the predictive ability of the constitutive model for the cumulative plastic strain of soil under intermittent cyclic loading is revealed. The constitutive model is compared with other existing models and the following main conclusions are drawn:

- By replacing the Newtonian dashpot in the generalized Kelvin model with the Abel dashpot, the creep constitutive equation of the fractional generalized Kelvin model is derived. Based on the viscoplastic theory, the strain response equation of the generalized Kelvin model is given, and a fractional generalized Kelvin model used to describe the stable and critical curve laws of soil cumulative plastic strain under cyclic loading is established.
- A nonlinear viscoplastic model considering damage is proposed, which can reflect the change law of the cumulative plastic strain failure curve of soil. It is connected with the fractional generalized Kelvin model to obtain the deformation constitutive model of soil under intermittent cyclic loading.
- Fitting parameters $\alpha$ and $\eta$ present a trend of decay and increase with the increase in loading stage *N*, respectively, which means that the soil gradually becomes denser, and its mechanical property gradually tends toward an ideal solid.
- By comparing with different models, the deformation constitutive models can well capture the development law of the soil cumulative plastic strain stable and failure curves, and can reflect the effect of confining pressure, frequency, dynamic stress amplitude and other factors on the cumulative plastic strain of the soil, while model parameters $\alpha$ and $\eta$ can characterize the mechanical properties of the soil.

## Data Availability

All data, models, and code generated or used during the study appear in the submitted article.
